# Hormonal intervention for the treatment of veterans with COVID-19 requiring hospitalization (HITCH): a multicenter, phase 2 randomized controlled trial of best supportive care vs best supportive care plus degarelix: study protocol for a randomized controlled trial

**DOI:** 10.1186/s13063-021-05389-0

**Published:** 2021-07-05

**Authors:** Nicholas G. Nickols, Matthew B. Goetz, Christopher J. Graber, Debika Bhattacharya, Guy Soo Hoo, Matthew Might, David B. Goldstein, Xinchen Wang, Rachel Ramoni, Kenute Myrie, Samantha Tran, Leila Ghayouri, Sonny Tsai, Michelle Geelhoed, Danil Makarov, Daniel J. Becker, Jun-Chieh Tsay, Melissa Diamond, Asha George, Mohammad Al-Ajam, Pooja Belligund, R. Bruce Montgomery, Elahe A. Mostaghel, Carlie Sulpizio, Zhibao Mi, Ellen Dematt, Joseph Tadalan, Leslie E. Norman, Daniel Briones, Christina E. Clise, Zachary W. Taylor, Jeffrey R. Huminik, Kousick Biswas, Matthew B. Rettig

**Affiliations:** 1 Radiation Oncology Service, VA Greater Los Angeles Healthcare System, Los Angeles, CA 90073 USA; 2 Infectious Diseases Section, VA Greater Los Angeles Healthcare System, Los Angeles, CA 90073 USA; 3 Division of Pulmonary and Critical Care, VA Greater Los Angeles Healthcare System, Los Angeles, CA 90073 USA; 4 Hugh Kaul Precision Medicine Institute, University of Alabama at Birmingham, Birmingham, USA; 5 Institute of Genomic Medicine, Columbia University Irving Medical Center, New York, USA; 6 Office of Research and Development, Veterans Health Administration, Washington, D.C. USA; 7 Division of Hematology-Oncology, VA Greater Los Angeles Healthcare System, Los Angeles, CA 90073 USA; 8 Division of Hematology-Oncology, VA New York Harbor Healthcare System, New York, USA; 9 Perlmutter Cancer Center, NYU Grossman School of Medicine, New York, USA; 10 Division of Pulmonary and Critical Care, VA New York Harbor Healthcare System, New York, USA; 11 Division of Hematology-Oncology, VA Puget Sound Healthcare System, Seattle, USA; 12 Geriatric Research Education and Clinical Care (GRECC), VA Puget Sound Health Care System, Seattle, USA; 13 VA Cooperative Studies Program Coordinating Center, Point, Perry, MD USA; 14 VA Cooperative Research Pharmacy Coordinating Center, Albuquerque, NM USA

**Keywords:** COVID-19, SARS-CoV-2, TMPRSS2, Androgen receptor, Androgen suppression, Coronavirus, Hormone therapy, Anti-androgen

## Abstract

**Background:**

Therapeutic targeting of host-cell factors required for SARS-CoV-2 entry is an alternative strategy to ameliorate COVID-19 severity. SARS-CoV-2 entry into lung epithelium requires the TMPRSS2 cell surface protease. Pre-clinical and correlative data in humans suggest that anti-androgenic therapies can reduce the expression of TMPRSS2 on lung epithelium. Accordingly, we hypothesize that therapeutic targeting of androgen receptor signaling via degarelix, a luteinizing hormone-releasing hormone (LHRH) antagonist, will suppress COVID-19 infection and ameliorate symptom severity.

**Methods:**

This is a randomized phase 2, placebo-controlled, double-blind clinical trial in 198 patients to compare efficacy of degarelix plus best supportive care versus placebo plus best supportive care on improving the clinical outcomes of male Veterans who have been hospitalized due to COVID-19. Enrolled patients must have documented infection with SARS-CoV-2 based on a positive reverse transcriptase polymerase chain reaction result performed on a nasopharyngeal swab and have a severity of illness of level 3–5 (hospitalized but not requiring invasive mechanical ventilation). Patients stratified by age, history of hypertension, and severity are centrally randomized 2:1 (degarelix: placebo). The composite primary endpoint is mortality, ongoing need for hospitalization, or requirement for mechanical ventilation at 15 after randomization. Important secondary endpoints include time to clinical improvement, inpatient mortality, length of hospitalization, duration of mechanical ventilation, time to achieve a normal temperature, and the maximum severity of COVID-19 illness. Exploratory analyses aim to assess the association of cytokines, viral load, and various comorbidities with outcome. In addition, TMPRSS2 expression in target tissue and development of anti-viral antibodies will also be investigated.

**Discussion:**

In this trial, we repurpose the FDA approved LHRH antagonist degarelix, commonly used for prostate cancer, to suppress TMPRSS2, a host cell surface protease required for SARS-CoV-2 cell entry. The objective is to determine if temporary androgen suppression with a single dose of degarelix improves the clinical outcomes of patients hospitalized due to COVID-19.

**Trial registration:**

ClinicalTrials.gov NCT04397718. Registered on May 21, 2020

## Background

SARS-CoV-2 recognizes host cell membrane proteins and relies upon their enzymatic activity to infect host cells. Recognition of the host surface protein ACE2 facilitates attachment of the virus to the host cell. However, entry of the virus into the host cell requires catalytic cleavage of the Viral Spike (S) protein (a process termed S protein priming) by the host cell membrane protein TMPRSS2 [1]. Thus, TMPRSS2 is required for viral entry and infection. TMPRSS2 is expressed within the nasal mucosa, respiratory sinuses, buccal mucosa, tracheal epithelia, bronchial epithelia, lung type 2 pneumocytes, and alveolar macrophages [2–4]. In addition to the aero-digestive tract, TMPRSS2 is highly expressed on prostate, kidney, and pancreatic epithelia [4].

The transcriptional regulation of the *TMPRSS2* gene has been extensively characterized, most rigorously within the prostate. The *TMPRSS2* gene is located on chromosome 21 and is under the control of the androgen receptor (AR). Binding of androgens (e.g., testosterone or dihydrotestosterone) to the AR results in homodimerization and translocation of the AR to the nucleus, where it binds to its cognate androgen response element in the regulatory regions of its target genes, and thereby regulates gene expression [5, 6]. It is firmly established that suppression of AR transcriptional activity through reduction in circulating androgens or direct antagonism of AR-androgen binding using AR competitive antagonists reduces expression and protein levels of TMPRSS2 within the prostate, as well as within prostate cancers [7].

Our team queried publicly available gene expression databases to identify FDA-approved drugs that downregulate TMPRSS2. Notably, anti-androgenic compounds and estrogens were among the strongest and most consistent downregulators of *TMPRSS2* expression, while androgens consistently led to upregulated *TMPRSS2* gene expression [8]. In other words, these studies support the notion that the AR *induces* TMPRSS2 expression, whereas estrogen receptor (ER) transcriptional activity is associated with *suppression* of TMPRSS2 expression.

Expression of TMPRSS2 also appears to be hormonally regulated within the lung and bronchial cells [9]. Androgens enrich AR binding at the TMPRSS2 enhancer and upregulate expression of *TMPRSS2* in human lung derived cells, in a fashion similarly to that found in the prostate [10]. Moreover, *AR*, *TMPRSS2*, and *ACE2* are co-expressed in human lung epithelial cells including alveolar and bronchial epithelial cells [9]. In mice, testosterone suppression reduces expression of both *TMPRSS2* and *ACE2* in lung bronchial cells*,* which was reversible upon testosterone repletion [9]. In vitro, anti-androgens reduced SARS CoV 2 infection in cultured human cells [9].

Based on the aforementioned data, it is hypothesized that drugs that interfere with AR driven transcription will reduce *TMPRSS2* and *ACE2* expression. FDA-approved drugs that block AR signaling include the GnRH analogs that reduce pituitary release of luteinizing hormone (LH), thereby potently suppressing testosterone production, and anti-androgens that interfere with binding of androgens to the AR. These drugs are predominantly used in the treatment of prostate cancer, have well known and generally tolerable side effect profiles, and exhibit reversibility of their biologic effects. We hypothesized that androgen suppression will reduce *TMPRSS2* expression in the target cells of SARS-CoV-2 and reduce the severity of COVID-19. This general hypothesis is supported by population level data from Italy that suggest a protective effect from androgen suppressive therapy against COVID-19 in prostate cancer patients [11].

Our objective is to investigate if temporary androgen suppression that reduces expression of TMPRSS2 will reduce severity of COVID-19 disease due to SARS-CoV-2 infection. Our specific hypothesis is that the LHRH antagonist degarelix reduces the composite endpoint of mortality, need for extended hospitalization, and/or need for invasive mechanical ventilation in patients hospitalized with COVID-19.

## Methods

### Study design

**Fig. 1:**
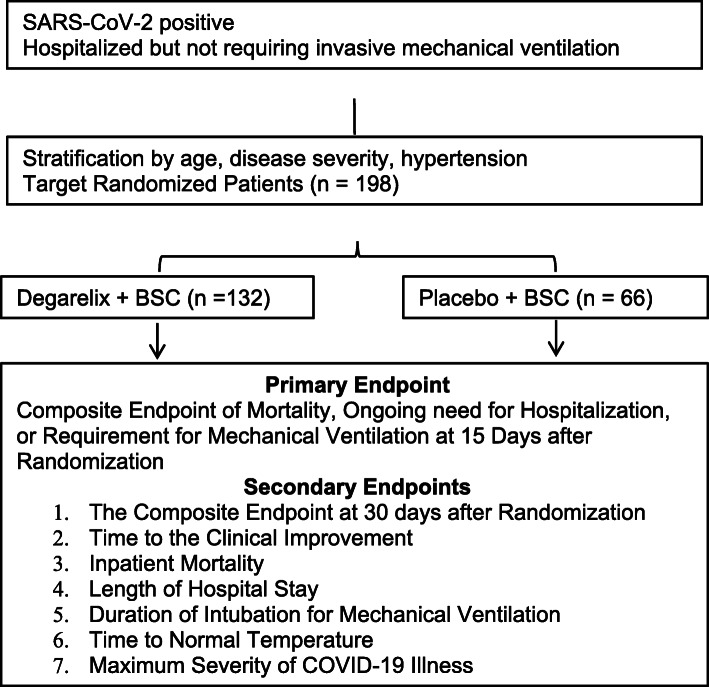
Trial schema

**Fig. 2:**
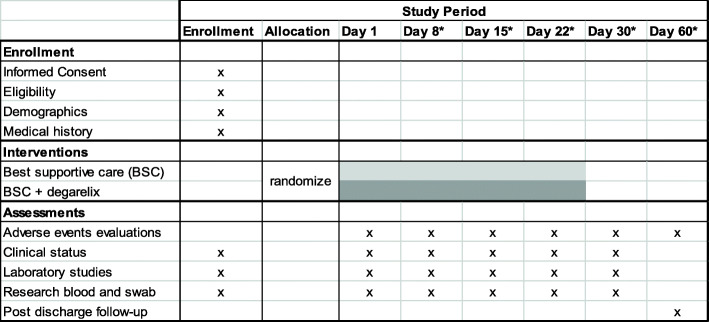
SPIRIT figure. Asterisk indicates if patient is still hospitalized

This is a randomized phase 2, placebo-controlled, double-blind clinical trial in 198 patients testing the efficacy of degarelix plus best supportive care versus placebo plus best supportive care for improvement in the clinical outcomes of male patients who have been hospitalized due to COVID-19 (see Fig. 1 for Schema, Fig. 2 for SPIRIT). Enrolled patients must have documented infection with SARS-CoV-2 based on a positive reverse transcriptase polymerase chain reaction result performed on a nasopharyngeal swab and have a severity of illness of level 3–5 (see Table 1) [12]. Stratification is by age (< 65 versus ≥ 65), history of hypertension, and disease severity score (3 versus 4 or 5), and centrally randomized 2:1 (degarelix: placebo). Best supportive care includes all treatments that would be applied irrespective of patient enrollment and includes but is not limited to supplemental oxygen, antibiotics, vasopressor support, peritoneal or hemodialysis, antibiotics, and intravenous fluids. The treatment landscape for COVID-19 is rapidly evolving. Accordingly, the clinical trial allows for updates to the allowed treatments. Remdesivir and convalescent plasma are allowed. New treatments will have a drug utilization report assessment prior to use to identify overlapping toxicities prior to use of a new treatment. The development of novel treatments and interventions for COVID-19 is monitored by site investigators as well as other study team members. Appropriate changes to allowed treatments and interventions are instituted accordingly. Patients randomized receive either a one-time dose of degarelix 240 mg (a 30 day depot) or placebo administered subcutaneously in the periumbilical area. Patients, investigators, and treating physicians are blinded to treatment assignment (i.e., double blinding) and randomization is accomplished centrally Unblinding for emergency situations is managed through a 24 h a day emergency call service managed by the VA CSP. Patients are followed for up to 60 days.

**Table 1:** 7-category ordinal scale of clinical status of hospitalized influenza patients

1: Not hospitalized with resumption of normal activities.	
2: Not hospitalized, but unable to resume normal activities	
3: Hospitalization, not requiring supplemental oxygen	
4: Hospitalization, requiring supplemental oxygen	
5: Hospitalization, requiring nasal high-flow oxygen therapy and/or noninvasive mechanical ventilation	
6: Hospitalization, requiring extracorporeal membrane oxygenation and/or invasive mechanical ventilation	
7: Death	

### Participants

The trial is open at the Greater Los Angeles, Manhattan, Brooklyn, Puget Sound, Phoenix, Central Arkansas, Long Beach, Miami, Philadelphia, Charleston, Memphis, St Louis, Dallas, and Houston Veterans Healthcare Systems. Inclusion and exclusion criteria are listed in Table 2. The trial is exclusive to males ≥ 18 who manifest symptoms and a disease severity that warrant hospitalization for supportive care but not invasive mechanical ventilation. In this patient population, the virus itself still drives the severity of the disease, whereas a hyperinflammatory response is thought to mediate ARDS, SIRS, and respiratory failure that underlie the severity of COVID-19 in critically ill, intubated ICU patients. Female patients are excluded in this study because female androgen suppression *reduces* endogenous estrogenic activity and thereby may induce TMPRSS2 expression, which in turn could exacerbate COVID-19.

**Table 2:** Inclusion and exclusion criteria

*Inclusion criteria* 1. Male Veterans admitted to a VA hospital. 2. Age ≥18 3. Hospitalized on an acute care ward with a diagnosis of COVID-19 contributing to hospitalization. 4. Positive RT-PCR assay for SARS-CoV-2 on a nasopharyngeal swab sample. 5. Severity of illness of level 3, 4, or 5 on the influenza severity scale at the time of randomization. 6. The subject (or legally acceptable representative if applicable) must provide written informed consent for the trial. *Exclusion criteria* 1. History of severe hypersensitivity to degarelix or any component of their respective formulation 2. History of congenital long QT syndrome or known history of prolonged QT interval OR Fridericia correction formula (QTcF) > 500 ms on electrocardiogram performed at screening. 3. Planned discharge within 24 h of treatment initiation. 4. Subject is planning to conceive or father children within the projected duration of the study, starting with the screening visit through 120 days after the last dose of study treatment. 1. Ongoing usage of a class IA or class III antiarrhythmic agent. At least 5 half-lives must elapse since any prior use of a class IA or III antiarrhythmic agent prior to administration of study drug. 5. Baseline electrolyte abnormalities of grade 3 or higher (based on CTCAE v5.0 criteria). Patients may be included if baseline electrolyte abnormalities are corrected to grade 2 or lower prior to study drug administration. 6. Myocardial infarction in the past 6 months, severe or unstable angina, or New York Heart Association (NYHA) class III or IV heart disease. 7. Enrollment in another investigational study within 30 days of day 1. 8. Known psychiatric or substance abuse disorder that would interfere with the requirements of the trial. 9. Child-Pugh Class C liver disease. 10. Use of any of the following hormonal agents: • Androgen receptor antagonists or agonists within 4 weeks of study enrollment, • Ketoconazole or abiraterone acetate within 2 weeks of study enrollment, • Estrogens or progestins within 2 weeks of study enrollment, • Herbal products that contain hormonally active agents within 2 weeks of study enrollment. • Any prior use of an LHRH analog unless a serum total testosterone measured within 30 days of study enrollment is ≥ 150 ng/dL. • Other hormonal agents listed in Appendix B within one day of study enrollment. 11. Unwilling or unable to comply with the study protocol. 12. Any condition, which in the opinion of the investigator, would preclude participation in the trial.	

### Study drug

Degarelix is an FDA-approved drug indicated for the treatment of prostate cancer. One loading dose of 240 mg given as two injections of 120 mg, a concentration of 40 mg/mL, administered subcutaneously in the abdominal region serves as a 30-day depot. As a LHRH antagonist, degarelix acts at the level the pituitary to rapidly reduce LH secretion, thereby decreasing testosterone production within the testes. A rapid reduction in circulating total testosterone to ≤ 50 ng/dl is achieved in the majority of patients within 24 h and virtually all patients in 2–3 days. Testosterone production recovers after the 30-day depot is cleared.

### Study endpoints

Primary, secondary, and exploratory endpoints are listed in Table 3. Briefly, the primary endpoint is a composite of mortality, need for ongoing hospitalization, or requirement for mechanical ventilation (including extracorporeal membrane oxygenation) at day 15 after randomization. This endpoint was selected because it is a clinically meaningful outcome for hospitalized patients. The secondary endpoints include time to clinical improvement, inpatient mortality, length of hospitalization, duration of intubation for mechanical ventilation, time to achieve a normal temperature, and the maximum severity of COVID-19 illness. Exploratory endpoints assess the association of D-dimer, IL-6, LDH, ferritin, total WBC, absolute neutrophil count, absolute lymphocyte count, testosterone, viral load (in nasopharyngeal swabs and blood), and various demographic and concurrent comorbidities with outcome. Additionally, for patients enrolled at the Greater Los Angeles, pharmacologic suppression of TMPRSS2 expression in the nasopharynx is also assessed via nasal swab, and the presence of anti-viral antibodies in blood are assessed over time.

**Table 3:** Endpoints and data analysis plan

Endpoint analyses	Statistical methods	SAS procedures
*Primary endpoint* A composite endpoint of mortality, hospital stay rate, and ECMO or mechanical intubation at 15 days after randomization	Pearson chi-square tests or Fisher exact tests Logistic regression adjusted for age, hypertension, and COPD	PROC FREQ PROC LOGISTC
*Secondary endpoints* 1. Time to the clinical improvement 2. Inpatient mortality 3. Length of hospital stay 4. Duration of intubation for mechanical ventilation 5. Time to normal temperature 6. Maximum severity of COVID-19 illness	1. Log-rank test, Kaplan-Meier curves Cox regression adjusted for age, hypertension, and COPD 2. Pearson chi-square tests or Fisher exact tests Logistic regression adjusted for age, hypertension, and COPD 3. Wilcoxon test; quantile regression 4. Wilcoxon test; Quantile regression 5. Log-rank test, Kaplan-Meier curves Cox regression adjusted for age, hypertension, and COPD 6. Pearson chi-square test; Cochran–Armitage trend test Proportional odds logistic regression	1. PROC LIFETEST PROC PHREG 2. PROC FREQ PROC GENMOD 3. PROC NPAR1WAY 4. PROC NPAR1WAY 5. PROC LIFETEST PROC PHREG 6. PROC FREQ PROC LOGISTC
*Exploratory analyses* Clinical prognostic factors Laboratory prognostic factors Viral load Cytokines levels and TMPRSS2 expression Germline genomic factors	1. Time to event data Log-rank tests, Kaplan-Meier curves, and Cox regressions 2. Categorical data Pearson chi-square tests and logistic regression 3. Interval data Student t/Wilcoxon tests, linear or quantile regressions 4. Longitudinal data Mixed-effect model repeated measure	1. PROC LIFETEST PROC PHREG 2. PROC FREQ PROC LOGISTC 3. PROC TTEST PROC NPAR1WAY 4. PROC Mixed
*AE/SAE analyses* 1. Incidence of AE/SAE 2. Frequency difference of AE and SAE 3. AE /SAE by relationship to the treatment 4. AE/SAE leading to premature discontinuation	*For All AE/SAE* Incidence rate estimation and testing Pearson chi-square tests or Fisher exact tests	PROC FREQ SAS Macros
*Other analyses* 1. Baseline characteristics 2. Disposition status 3. Adherence 4. Site Effect	1. Student t or Wilcoxon tests, Pearson chi-square or Fisher exact tests 2. Pearson chi-square tests 3. Pearson chi-square tests 4. Pearson chi-square tests, Student t or Wilcoxon tests, log-rank test	1. PROC TTEST, PROC FREQ, PROC NPAR1WAY 2. PROC FREQ 3. PROC FREQ 4. PROC FREQ PROC LIFETEST PROC NPAR1WAY

### Safety evaluations/monitoring

Safety monitoring is conducted at scheduled study time points and through spontaneous reports from participants to evaluate adverse events (AEs). Adverse events of special interest include cardiac arrhythmias and thromboembolic events that may result from androgen suppression. Specifically, cardiac arrhythmias and thromboembolic complications of grades 3–5 (CTCAE v5.0). Non-serious adverse events related to the study intervention are reported to the sponsor through the electronic data capture (EDC) system. Expedited reporting of AEs of special interest (grades 3–5) and serious adverse events (SAEs) undergo daily review and reports generated for regular planned teleconference calls among the study investigators. Additionally, the VA Clinical Sciences Research and Development (CSR&D) centralized Data Monitoring Committee (DMC) will receive and review study reports monthly. Predetermined toxicity thresholds have been established as quality tolerance limits for statistical analysis. Stopping rules are applied if unbalanced toxicity signals are detected in the active arm at an α-level of 0.01.

### Statistical considerations

We assumed an effect size for the primary endpoint of 42%. A total of 186 evaluable patients (i.e., 124 evaluable patients in the degarelix group and 62 evaluable patients in the placebo group) have 90% power of detecting the expected effect size using a two-sided two proportion test with a significance level of 0.05. Based on an assumed 5% attrition rate, 198 patients will be required (i.e., 132 in the degarelix group and 66 in the placebo group). The primary analysis of the study will be performed on the primary endpoint on the intention to treat population. Secondary endpoint analyses will be adjusted for multiplicity with a α-level of 0.0083 for each endpoint. The endpoints and specific statistical methods used for each are listed in Table 3. A mid-term interim analysis of the primary endpoint is planned after half of the patients complete the study. If the mid-term analysis of the primary endpoint indicates that the null hypothesis can be rejected with a boundary value of 2.77 at an α-level of 0.006 or accepted with a boundary value of 0.44 based on O’Brien and Fleming criteria, the study will be recommended for trial termination for efficacy or futility. Additionally, once the toxicity markers reach a threshold of 25%, a statistical analysis of the toxicity will be performed. If the one group has a significantly higher rate of toxicity compared to the placebo group at an α-level of 0.01, then results will be reported to the DMC for a recommendation for trial termination.

### Trial status

The current protocol version number is 5.5., February 9, 2021. Enrollment began July 6, 2020. Estimated date of recruitment completion is July 6, 2021.

## Discussion

Pre-clinical and correlative data in humans suggest that anti-androgenic therapies can reduce the expression of TMPRSS2 on lung epithelium. Accordingly, we hypothesized that therapeutic targeting of androgen receptor signaling will suppress viral infection and thereby ameliorate the severity of symptomatic COVID-19. The purpose of this trial is to determine if temporary androgen suppression improves the clinical outcomes of patients who are hospitalized to an acute care ward due to COVID-19.

Degarelix was selected among the numerous FDA-approved drugs that target the AR analogs due to its rapid effect on circulating testosterone, safety profile, and availability. In contrast, LHRH agonists (e.g., leuprolide) achieve testosterone suppression over 2–3 weeks and induce a transient increase in serum testosterone, which could exacerbate the severity of the infection. The potent androgen receptor antagonists (e.g., enzalutamide and apalutamide) achieve steady state in the serum over approximately 4 weeks. Of all the FDA-approved agents that inhibit AR signaling, degarelix exhibits the most rapid and robust effects. Degarelix has over a decade of post-marketing safety information collected from its use in patients with prostate cancer. Documented side effects are often predictable in terms of their onset, duration, and severity. These side effects include fatigue, hot flashes, reduced libido, reduced fertility, and result directly from testosterone suppression. These can reduce quality of life but are not life threatening and are reversed upon testosterone recovery. Most of the more severe side effects associated with androgen suppression occur after long term use. These include potential worsening of preexisting cardiovascular disease, reduced bone mineral density, loss of lean muscle mass, hypercoagulability, and prolongation of the QT/QTc interval. Most patients are expected to recover testosterone within a few months after the single depot of degarelix, limiting the risks associated with long term androgen suppression. For the purposes of safety monitoring, we consider any grade 3 or higher CTCAEv5 toxicities associated with hypercoagulation or arrhythmias to be of special interest given the baseline elevated risks of hypercoagulability in hospitalized patients with COVID-19 and the risks of torsade de pointes associated with long QT/QTc intervals. We also chose to exclude patients with congenital long QT syndrome or known history of prolonged QT interval and require a screening electrocardiogram for QT interval corrected by the Fridericia correction formula (QTcF) > 500 ms.

Pharmacologic inhibition of TMPRSS2 with camostat mesilate prevents SARS-CoV-2 entry into cultured human lung cells [1]. However, a randomized trial of camostat mesilate versus placebo in patients hospitalized for COVID19 demonstrated no clinical improvement, ICU admission, or mortality [13].

In addition to our group, others are testing AR antagonism as a therapeutic strategy for hospitalized patients with COVID-19. The COVIDENZA trial led by the University of Gothenburg is an ongoing randomized trial of enzalutamide plus standard of care versus standard of care in hospitalized patients with COVID-19 [14]. A trial in the outpatient setting recently reported that antiandrogen treatment accelerated viral clearance as compared to placebo [15]. Whether or not AR antagonism should be considered as a therapeutic strategy for patients hospitalized with COVID-19 will depend on the results of this and related trials.

## Data Availability

Data sharing is not applicable to this protocol manuscript as no datasets were generated or analyzed.

## Abbreviations

ACE2: Angiotensin-converting enzyme 2
AR: Androgen receptor
COPD: Chronic obstructive pulmonary disease
CSR&D: Clinical Sciences Research and Development
DMC: Data Monitoring Committee
ECMO: Extracorporeal membrane oxygenation
ER: Estrogen receptor
FDA: Food and Drug Administration
GnRH: Gonadotropin-releasing hormone
LDH: Lactate dehydrogenase
LHRH: Luteinizing hormone-releasing hormone
SARS-CoV-1: Severe acute respiratory syndrome coronavirus 1
SARS-CoV-2: Severe acute respiratory syndrome coronavirus 2
S protein: Spike protein
TMPRSS2: Transmembrane protease, serine 2
WBC: White blood cell count

## References

[1] Hoffmann M, Kleine-Weber H, Schroeder S, Krüger N, Herrler T, Erichsen S, Schiergens TS, Herrler G, Wu NH, Nitsche A, Müller MA, Drosten C, Pöhlmann S (2020). SARS-CoV-2 Cell entry depends on ACE2 and TMPRSS2 and is blocked by a clinically proven protease inhibitor. Cell..

[2] Bertram S, Heurich A, Lavender H, Gierer S, Danisch S, Perin P, Lucas JM, Nelson PS, Pöhlmann S, Soilleux EJ (2012). Influenza and SARS-coronavirus activating proteases TMPRSS2 and HAT are expressed at multiple sites in human respiratory and gastrointestinal tracts. PLoS ONE..

[3] Yamaya M, Shimotai Y, Hatachi Y, Lusamba Kalonji N, Tando Y, Kitajima Y, Matsuo K, Kubo H, Nagatomi R, Hongo S, Homma M, Nishimura H (2015). The serine protease inhibitor camostat inhibits influenza virus replication and cytokine production in primary cultures of human tracheal epithelial cells. Pulm Pharmacol Ther..

[4] Vaarala MH, Porvari KS, Kellokumpu S, Kyllönen AP, Vihko PT. Expression of transmembrane serine protease TMPRSS2 in mouse and human tissues. J Pathol. 2001;193(1):134–40. 10.1002/1096-9896(2000)9999:9999<::AID-PATH743>3.0.CO;2-T. 10.1002/1096-9896(2000)9999:9999<::AID-PATH743>3.0.CO;2-T 11169526

[5] Clinckemalie L, Spans L, Dubois V, Laurent M, Helsen C, Joniau S, Claessens F (2013). Androgen regulation of the TMPRSS2 gene and the effect of a SNP in an androgen response element. Mol Endocrinol..

[6] Chen Z, Song X, Li Q, Xie L, Guo T, Su T, et al. Androgen receptor-activated enhancers simultaneously regulate oncogene TMPRSS2 and lncRNA PRCAT38 in prostate cancer. Cells. 2019;8(8). 10.3390/cells8080864. 10.3390/cells8080864 PMC6721761 31405024

[7] Lin B, Ferguson C, White JT, Wang S, Vessella R, True LD, Hood L, Nelson PS (1999). Prostate-localized and androgen-regulated expression of the membrane-bound serine protease TMPRSS2. Cancer Res..

[8] Wang X, Dhindsa R, Povysil G, Zoghbi A, Motelow J, Hostyk J, et al. Transcriptional inhibition of host viral entry proteins as a therapeutic strategy for SARS-CoV-2. 2020. 10.20944/preprints2020030360.v1.

[9] Qiao Y, Wang X-M, Mannan R, Pitchiaya S, Zhang Y, Wotring JW, et al. Targeting transcriptional regulation of SARS-CoV-2 entry factors ACE2 and TMPRSS2. Proc Natl Acad Sci U S A. 2020;118(1):e2021450118. 10.1073/pnas.2021450118. Online ahead of print. 10.1073/pnas.2021450118 PMC7817128 33310900

[10] Mikkonen L, Pihlajamaa P, Sahu B, Zhang F-P, Jänne OA (2010). Androgen receptor and androgen-dependent gene expression in lung. Mol Cell Endocrinol..

[11] Montopoli M, Zumerle S, Vettor R, Rugge M, Zorzi M, Catapano CV, Carbone GM, Cavalli A, Pagano F, Ragazzi E, Prayer-Galetti T, Alimonti A (2020). Androgen-deprivation therapies for prostate cancer and risk of infection by SARS-CoV-2: a population-based study (n = 4532). Ann Oncol..

[12] Wang Y, Fan G, Horby P, Hayden F, Li Q, Wu Q (2019). Comparative outcomes of adults hospitalized with seasonal influenza A or B virus infection: application of the 7-category ordinal scale. Open Forum Infect Dis.

[13] Gunst JD, Staerke NB, Pahus MH, Kristensen LH, Bodilsen J, Lohse N, Dalgaard LS, Brønnum D, Fröbert O, Hønge B, Johansen IS, Monrad I, Erikstrup C, Rosendal R, Vilstrup E, Mariager T, Bove DG, Offersen R, Shakar S, Cajander S, Jørgensen NP, Sritharan SS, Breining P, Jespersen S, Mortensen KL, Jensen ML, Kolte L, Frattari GS, Larsen CS, Storgaard M, Nielsen LP, Tolstrup M, Sædder EA, Østergaard LJ, Ngo HTT, Jensen MH, Højen JF, Kjolby M, Søgaard OS (2021). Efficacy of the TMPRSS2 inhibitor camostat mesilate in patients hospitalized with COVID-19-a double-blind randomized controlled trial. EClinicalMedicine..

[14] Welén K, Överby AK, Ahlm C, Freyhult E, Robinsson D, Henningsson AJ, Stranne J, Bremell D, Angelin M, Lindquist E, Buckland R, Carlsson CT, Pauksens K, Bill-Axelsson A, Akre O, Ryden C, Wagenius M, Bjartell A, Nilsson AC, Styrke J, Repo J, Balkhed ÅÖ, Niward K, Gisslén M, Josefsson A (2021). COVIDENZA - a prospective, multicenter, randomized PHASE II clinical trial of enzalutamide treatment to decrease the morbidity in patients with Corona virus disease 2019 (COVID-19): a structured summary of a study protocol for a randomised controlled trial. Trials..

[15] Cadegiani FA, McCoy J, Gustavo Wambier C, Vaño-Galván S, Shapiro J, Tosti A (2021). Proxalutamide significantly accelerates viral clearance and reduces time to clinical remission in patients with mild to moderate COVID-19: results from a randomized, double-blinded, placebo-controlled trial. Cureus..

